# Supplementary material for: Early surgical treatment using regional clinical pathways to reduce the length of postoperative hospital stay in hip fracture patients: A retrospective analysis using the Japanese Diagnosis Procedure Combination database
Source: PLoS One. 2024 Jul 31;19(7):e0282766. doi: 10.1371/journal.pone.0282766 (PMC11290638; doi:10.1371/journal.pone.0282766)
Supplement: Attachment — Submitted filename: R1-2_response_to_reviewers.docx [file pone.0282766.s001.docx]

**Reviewer 1**

1. Introduction section:
   Line 51-53: these statistics belong to which country?? Even the reference refers to Japanese statistics, the text itself is not clear.

**Response:** We apologize for the lack of clarity. The statistics are for Japan; this has now been specified in the revised manuscript (Line 53).

2. Results section:
   The prevalence of preexisting comorbidities is strongly affecting the results especially the length of stay LOS,　so attributing this reduction of LOS to only the implementation of regional clinical pathways is not accurate, this is a selection bias. Even so, in the limitation section, no referral to this point, thus both early and delayed surgery groups are not the same in prevalence of comorbidities which typically will influence the results. So, I want a clear statement about this point.

**Response:** Thank you for your valuable insights. We expected that comorbidities at admission would affect the length of hospital stay. In fact, Table 1 shows that the early surgery group had fewer comorbidities on admission than did the delayed surgery group. On the other hand, Table 3 shows the effect of early surgery and the regional clinical pathway on the length of hospital stay after adjusting for admission comorbidities and postoperative complications as background factors. However, as you pointed out, there may have been a selection bias because patients with fewer comorbidities on admission could have received earlier surgery. We have included this point as a limitation of the study in the revised manuscript (Lines 318-322).

3. Conclusion section:
   I think should be rephrased, with clearer strong statement, with rearrangement of sentences. First sentences, lines 300~303 just describe a fact which is not discovered by the authors nor first reported. I suggest starting the second sentence; i., e about relation between early surgery and rate of complication should be the striking finding that make the clear impression with the reader.
   **Response:** Thank you for your valuable suggestions. We have modified the conclusion section per your advice (Lines 327-335).

**Reviewer 2**

1. Abstract:
   Hypothesis is missing
   **Response:** We apologize for the missing hypothesis. It has now been added in the Abstract as follows:

“We hypothesized that early surgery with implementation of a regional clinical pathway would be effective in reducing postoperative LOS and complications in hip fracture patients.” (Lines 30-32)

2. Introduction
   Hypothesis is missing.
   **Response:** We apologize for the missing hypothesis. It has now been added in the Introduction (Lines 94-96).
3. Methods:
   Please explain the regional pathways.

**Response:** Thank you for your advice. An explanation of a reginal clinical pathway has been added in the Materials and Methods section (Factor setting subsection) as follows:
“A regional clinical pathway is established to be shared and used in multiple coordinating medical institutions authorized to treat patients with health insurance coverage or nursing care facilities following patient transfer or discharge from the administering hospital. It records disease names, symptoms at the time of hospitalization, scheduled medical examinations, the average duration of patient transfers, contents of medical treatment after patient transfers, average time course of discharge from the coordinating medical institutions, release standard expected at the time of discharge, and other required items.” (Lines 131-138).

4. There are more comorbidities in the delayed group. Thus, the increased postoperative complication rate is not surprising.

**Response:** Thank you for pointing this out. The prevalence of postoperative complications in the delayed surgery group was as expected, and it was used to adjust for background factors when identifying the effect on the postoperative hospital stay, as described in Table 3.

This is also true for variables such as comorbidities at admission in Table 1, which were also used to adjust for background factors. In the Statistical analysis section, we have specified that the prevalence of postoperative complications was used to adjust for background factors in the multiple logistic regression analysis (Lines 151-152).

5. Table 2 – What is meant by cardiovascular disease as a complication? Heart attack?

**Response:** We apologize for the lack of clarity. Cardiovascular disease included ischemic heart disease, arrhythmias, heart failure, and hypertension. We have specified this in the Materials and Methods section (Factor setting subsection) and the legend for Table 2 (Lines 143-144 and 195-196).

6. The authors need to clarify the regional pathways. The 3-week length of stay is long and not typical in other countries such as the USA. Further, it is unclear to me how the LOS of a transferred patient is shorter. Are they being transferred the same day?
   **Response:** We apologize for the lack of clarity. First, we have added a description of the regional clinical pathway in the Materials and Methods section (Factor setting subsection) (Lines 131-138).

Second, a recent study reported that the average length of hospital stay for hip fracture patients in Japan is 31 days (Maki et al, Impact of the Comorbidity Polypharmacy Score on Clinical Outcome in Patients with Hip Fracture undergoing surgery Using Real-World Data, Annals of Clinical Epidemiology, 2023, 5, 3, p. 88-94,). Therefore, a total LOS of 30-37 days, as documented in the present study, is reasonable.

Although we do not have data to show whether or not the transferred patients were transferred to the hospital for surgery on the same day, we believe that most of them were transferred on the same day or 1 day after their injury. We speculate that the reduced LOS for transferred patients was possible because of good cooperation between small hospitals that cannot perform surgeries and big hospitals that perform surgery. In general, they cooperate well with each other to facilitate smooth transfers and surgeries for patients in Japan. Therefore, the early surgery group had more patients transferred from another hospital at admission, and this factor effectively reduced the postoperative LOS. We have added this explanation in the Discussion section (Lines 305-311).

7. The data needs to be analyzed accounting for delayed surgery not only for more than 48h but also for the absolute timeframe. It is not surprising that the LOS of a patient who has surgery in 5 days may be longer than the one in 3 days.
   **Response:** Thank you for your valuable insights. In this study, the effect of early surgery on postoperative hospital stay was calculated using logistic regression analysis with an adjusted model. The results showed that early surgery within 2 days was effective in reducing postoperative LOS by 2.58 days, which is considered to be a reduction in LOS excluding the waiting period for surgery. On the other hand, we could not analyzed the effect on LOS using an absolute timeframe including the waiting period. This has been mentioned as a limitation of the study in the Discussion section (Lines 321-322).
8. There is a lower rate of anemia in the delayed group which is surprising as more blood loss would be expected with an untreated femur fracture. The discussion should provide a better explanation.
   **Response:** Thank you for your comments. This result is attributed to the fact that the early surgery group included more peritrochanteric fractures, which result in greater blood loss than do femoral neck fractures, which were more common in the delayed surgery group. We have discussed this point in the Discussion section (Lines 256-263).
9. The main result of this study is the effect of regional clinical pathways on LOS. This needs to be explained more detail and should be the main focus of this manuscript. What are these pathways? Are they different from international pathways?
   **Response:** Thank you for your comments. We have added a subsection titled “Contribution of regional clinical pathways and other factors to LOS reduction” in the Discussion section in order to emphasize the effect of regional clinical pathways on LOS reduction (Lines 292-304).

We have also added a description of the regional clinical pathway in the Materials and Methods section (Factor setting subsection) (Lines 131-138).

10. Table 1 – There is a significant difference between postoperative days until rehabilitation initiated although both are listed as one day. Please explain and add decimals.
    **Response:** Thank you for pointing this out. We apologize for the error; these values were the same because of an error in the statistical software settings. We have recalculated and corrected the values. Postoperative days until initiation of rehabilitation were 1.61 and 1.40 days in the early and delayed surgery groups, respectively. The table has been rectified.

**Note:**

We kindly ask PLOS ONE to update the grant information in Financial Disclosure as following:

*Currently*- HS received a Grant-in-Aid for Scientific Research [grant numbers (C) 20K09421] from the Japan Society for the Promotion of Science (JSPS). <https://www.jsps.go.jp/j-grantsinaid/> The funders had no role in study design, data collection and analysis, decision to publish, or preparation of the manuscript.

*Update*- HS received a Grant-in-Aid for Scientific Research [grant numbers (C) 20K09421 and (C) 23K08644] from the Japan Society for the Promotion of Science (JSPS). <https://www.jsps.go.jp/j-grantsinaid/> The funders had no role in study design, data collection and analysis, decision to publish, or preparation of the manuscript.
